# Sleep patterns of Japanese preschool children and their parents: implications for co-sleeping

**DOI:** 10.1111/apa.12203

**Published:** 2013-03-06

**Authors:** Sachiko Iwata, Osuke Iwata, Toyojiro Matsuishi

**Affiliations:** Centre for Developmental and Cognitive Neuroscience, Department of Paediatrics and Child Health, Kurume University School of MedicineFukuoka, Japan

**Keywords:** Actigraphy, Co-sleeping, Parent–child interaction, Preschool children, Sleep pattern

## Abstract

**Aim:**

The aim of this study was to investigate the direct relationship of sleep schedule and sleep quality variables between healthy preschool children and their parents, focusing on the influence of the difference in bedtime between each other.

**Methods:**

Forty-seven Japanese 5-year-old children and their primary parent were studied. The parents completed questionnaires including the Epworth Sleepiness Scale and Pittsburgh Sleep Quality Index. The children wore an actigraph for one week.

**Results:**

Although sleep patterns of children were generally independent of their parents, late sleep end time and bedtime of children were associated with parents' late sleep end time on weekends. For 87% of children and parents who shared a bedroom, sleep quality was negatively affected by a shorter difference in bedtimes between child and parent, but not by co-sleeping.

**Conclusion:**

Sleep behaviours of parents can influence those of their children. For parents and children who share a bedroom, the timing of bedtime rather than co-sleeping may be a key factor in modulating sleep patterns. Trying to get children asleep and subsequently falling asleep at a similar time may disturb parents' sleep quality, which may subsequently affect that of their children.

## Key notes

- Sleep patterns of preschool children can be influenced by sleep behaviours of their parents.
- For Japanese parents and children who share a bedroom, the timing of bedtime rather than co-sleeping appeared to be an important determining factor of sleep quality.
- Falling asleep at a similar time as their children may disturb parents#x2019; sleep quality, which may subsequently influence that of their children.

## Introduction

Sleep patterns and sleep behaviours of children are influenced by a number of factors, including biological, cultural, social and family backgrounds. Despite increasing evidence suggesting the pivotal influence of parental and environmental factors 1,2, only a few studies have specifically examined the direct association between sleep patterns in healthy, typically developing children and their parents. One study that recruited children with a wide age range (3 years of age on average) observed the synchrony of maternal chronotype and children's sleep patterns, suggesting the interplay of sleep habits between mothers and young children 3. In contrast, another study that compared sleep schedule variables between children in their early teens and their parents demonstrated a lack of reciprocal relationships 4. These conflicting findings might be attributed to the difference in children's age and subsequent alteration in sleep arrangements.

Of several environmental factors, the influence of co-sleeping has been widely recognized. Co-sleeping has been linked with a range of sleep problems such as frequent awakenings and difficulty in getting back to sleep thereafter 5–8. However, previous studies have not fully addressed differences in co-sleeping arrangements, such as the type and size of the bed and bedroom or parental proximity and body contact as the children fall asleep. These factors may directly affect the interplay of sleep habits between children and their parents.

In addition to environmental factors, cross-cultural backgrounds deserve consideration when assessing the influence of co-sleeping on sleep quantity and quality. A comparative study of co-sleeping and solitary sleeping in young children in Japan and the United States demonstrated that co-sleeping was associated with overall stressful sleep problems in the United States sample but not in the Japanese sample 9. This suggests the possibility that the adverse influence of co-sleeping appears mainly in conjunction with certain types of sleep arrangements and/or sleep habits. In Asian countries, co-sleeping occurs as a natural extension of bedroom sharing. For example, Japanese traditional beds, namely futons, are arranged on the floor in rows without clear boundaries between family members. Therefore, it is possible that the time when parents fall asleep (i.e. at the same time as their children or at a different time) can be a more important independent variable of their sleep patterns rather than the difference between co-sleeping and mere bedroom sharing.

To investigate the direct relationship of sleep schedule and sleep quality variables between Japanese preschool children and their parents, we performed a study in a cohort of healthy, typically developing Japanese preschool children and their parents, focusing on the influence of the difference in bedtime between the two.

## Method

This study was conducted according to the guidance and approval of the ethics committee of Kurume University School of Medicine, and parental informed consent was obtained.

We used the same data set as our previous studies that investigated the sleep habits of healthy five-year-old children using actigraphy 10,11. These studies demonstrated the dependence of sleep patterns of young children on both intrinsic and extrinsic variables, such as gender, childcare and other parental preferences.

### Participants

Details of the study population and the data acquisition protocol have been described elsewhere 10–12. In brief, the study population comprised 47 5-year-old children (27 males and 20 females) and their parents, who were recruited from two day-care nurseries in the city of Kurume.

### Data collection

Participating children wore an actigraph watch on their wrist from a Friday afternoon to the next Friday afternoon (Ambulatory Monitoring, Ardsley, NY, USA). Primary parents, who spent more time with the child around night time (46 mothers and one father), submitted the child's daily sleep log for the corresponding period for actigraphic validation. The Epworth Sleepiness Scale (ESS) and the Pittsburgh Sleep Quality Index (PSQI) were used to assess daytime sleepiness and global sleep quality/disturbances for parents, respectively. The ESS scores >10 and PSQI scores >5 were regarded as indicating excessive daytime sleepiness and poor sleep, respectively 13,14. Because the PSQI is likely to reflect the information during weekdays 12, parents were asked to report their sleep–wake patterns on the weekend. Finally, the parents completed brief questionnaires enquiring about their sleep satisfaction, the presence of sleep problems in their child and the sleep arrangement for their child.

### Data analysis

Actigraphic data of the children were analysed to provide the bedtime, sleep onset time, sleep end time, sleep period, sleep latency and sleep efficiency (fraction of the sleep period excluding waking after sleep onset relative to the sleep period) using an established algorithm 15. Because sleep variables of young children are affected by nursery attendance and weekday–weekend difference 10, variables were assessed for weekdays and the weekend, respectively, and the weekend was defined as a nursery off day (14 participants attended the nurseries on Saturdays; see reference 12 for detail). Pearson's product–moment correlations and Spearman's rank correlations were used to assess the correlations in sleep variables between the children and parents.

To assess the influence of sleep arrangements on sleep pattern, participants were grouped into four categories according to the sleep arrangement of the children: (i) solitary sleeping (solitary sleeping group), (ii) sharing bedroom with family members other than parents (separate bedroom group), and (iii) sharing bedroom with parents (bedroom sharing group). The last group was further divided into co-sleeping participants (co-sleeping subgroup) and others (non co-sleeping subgroup). Sleep variables were compared between groups using an analysis of variance and Fisher's least significant difference test. Finally, in the bedroom sharing group, the difference in bedtime between children and parents (ΔBT) was calculated as the parent's bedtime deducted from that of the child, which was compared with sleep variables using Pearson's product–moment correlations and Spearman's rank correlations.

Because of the exploratory nature of the study, p-values are shown without correcting for multiple comparisons.

## Results

All children completed the actigraphic data collection. The mean age of the parents was 35.4 years (range: 26–46 years), and none was engaged in a night job. Demographic data of sleep variables for the children and their parents are shown in the Table1. Sixty-six percentage of parents were satisfied with their sleep, whereas 13% and 34% showed abnormally high ESS (>10) and PSQI (>5) scores, respectively. Thirty-six percentage of children were considered by their parent to have sleep problems such as long sleep latencies (n =* *10), unfixed sleeping places (n =* *3), irregular sleep patterns (n =* *2) and night awakenings (n =* *2).

**Table 1:** Sleep variables measured in parents and children

Sleep variables		Parents	Children	p
Weekday				
Bedtime	(h)	23.1 (1.3)	21.5 (0.6)	<0.001
Sleep end time	(h)	6.4 (0.9)	7.1 (0.5)	<0.001
Sleep period	(h)	7.0 (1.2)	9.5 (0.5)	<0.001
Weekend				
Bedtime	(h)	23.5 (1.3)	22.0 (0.9)*	<0.001
Sleep end time	(h)	7.6 (1.7)*	7.4 (0.7)*	ns
Sleep period	(h)	8.1 (1.3)*	9.3 (0.8)	<0.001
Sleep latency	(min)	16.6 (13.8)	8.8 (4.5)	<0.001
Sleep efficiency	(%)	–	90.3 (5.6)	–
Sleep problems identified by parents		–	17 (36)†	–
ESS score		3.9 (5.0)	–	–
ESS score >10		6 (13)†	–	–
PSQI score		4.6 (2.2)	–	–
PSQI score >5		16 (34)†	–	–
Sleep satisfaction		31 (66)†	–	–

For parents, the sleep end time was later and the sleep period was longer on the weekend than on weekdays. Children's sleep schedules were later on the weekend than on weekdays; no difference in the sleep period was noted. Parents had a later bedtime, an earlier sleep end time and a shorter sleep period than their children on weekdays.

p values are from the analysis of variance between parents and children.

* Significant difference between weekdays and weekends.

† Data are shown as mean (standard deviation) or number of incidences (%).

Sleep variables were obtained differently for the children (actigraphy) and the parents (questionnaires).

ESS, Epworth Sleepiness Scale. PSQI, Pittsburgh Sleep Quality Index. ns, not significant.

### Relationship of sleep variables between children and parents

A late sleep end time of parents on the weekend was associated with a late sleep end time on weekdays and the weekend (p <* *0.01 and 0.05, respectively) and a late bedtime on weekdays (p <* *0.05) of the children (Table2). However, bedtime and the sleep period were not associated between the children and parents during the week. Low sleep efficiency of the children on weekdays was associated with an early bedtime (p <* *0.05) and a long sleep period (p <* *0.01) of the parents on weekdays. In contrast, abnormally high ESS scores of parents were associated with children's early bedtime on the weekend and high sleep efficiency on weekdays (both p <* *0.05).

**Table 2:** Correlation coefficients of sleep variables between parents and children

		Child's sleep variable							
		Bedtime		Sleep end time		Sleep period		Sleep efficiency	
Parental sleep variables		Weekday	Weekend	Weekday	Weekend	Weekday	Weekend	Weekday	Weekend
Bedtime	Weekday	0.10	−0.02	0.20	0.23	0.05	0.25	0.37*	0.17
	Weekend	0.15	0.27	0.20	0.16	−0.03	−0.16	0.11	0.18
Sleep end time	Weekday	0.15	0.10	0.39**	0.18	0.16	0.04	0.03	−0.01
	Weekend	0.34*	0.28	0.48**	0.38*	−0.02	0.03	0.15	0.07
Sleep period	Weekday	0.02	0.04	0.03	−0.18	0.00	−0.23	−0.40**	−0.35*
	Weekend	0.28	0.07	0.41**	0.30	0.02	0.19	0.08	−0.09
ESS score >10		−0.17	−0.32*	−0.06	−0.21	0.15	0.18	0.33*	0.20
PSQI score >5		−0.11	−0.10	−0.12	0.00	0.02	0.12	0.10	−0.03
Sleep satisfaction		0.06	−0.03	0.20	−0.04	0.16	−0.02	−0.31*	−0.26

*p < 0.05 and **p < 0.01, from Pearson's product–moment correlations or Spearman's rank order correlations.

ESS = Epworth Sleepiness Scale; PSQI = Pittsburgh Sleep Quality Index.

### Influence of sleep arrangement on sleep variables

Three children did not have a regular sleeping place and were excluded from further analysis because of their highly heterogeneous backgrounds. No child was assigned to the solitary sleeping group, whereas 3 and 41 children were assigned to the separate bedroom group (all children shared a bedroom with their siblings) and bedroom sharing group, respectively. Twenty-three children in the bedroom sharing group were co-sleeping (co-sleeping subgroup) and 18 were not (non co-sleeping subgroup) (Table3).

**Table 3:** Influence of co-sleeping on sleep variables measured for parents and children

		Parents			Children		
		Separate bedroom group (n = 3)	Bedroom sharing group		Separate bedroom group (n = 3)	Bedroom sharing group	
Sleep variables			Non co-sleeping (n = 18)	Co-sleeping (n = 23)		Non co-sleeping (n = 18)	Co-sleeping (n = 23)
Weekday bedtime	(h)	25.2 (1.4)	23.1 (1.3)*	22.9 (1.1)*	21.4 (0.8)	21.4 (0.5)	21.7 (0.6)
Weekday sleep end time	(h)	7.2 (0.8)	6.1 (1.1)	6.5 (0.6)	7.3 (0.9)	7.0 (0.4)	7.2 (0.5)
Weekday sleep period	(h)	6.3 (0.6)	6.8 (1.2)	7.1 (1.0)	9.9 (0.5)	9.5 (0.5)	9.4 (0.5)
Sleep latency	(min)	28.4 (12.7)	17.3 (14.7)	15.0 (13.2)	7.6 (2.0)	8.6 (4.4)	9.0 (5.0)
Sleep efficiency	(%)	–	–	–	94.4 (3.8)	90.5 (5.7)	89.8 (5.9)
Sleep problem^†^		–	–	–	1 (33)	7 (39)	7 (30)
ESS score >10^†^		1 (33)	1 (6)	4 (17)	–	–	–
PSQI score >5^†^		2 (67)	8 (44)	6 (26)	–	–	–
Sleep satisfaction^†^		2 (67)	9 (50)	18 (78)	–	–	–
ΔBT	(h)	3.8 (1.3)	1.7 (1.4)*	1.3 (1.2)**	–	–	–

Data are shown as mean (standard deviation) or number of incidences (%)^†^. Sleep variables are obtained differently for the children (actigraphy) and the parents (questionnaires).

*p < 0.05 and **p < 0.01, compared with no bedroom shared with children from unpaired analysis of variance and post hoc with Bonferroni correction.

ESS = Epworth Sleepiness Scale; PSQI = Pittsburgh Sleep Quality Index; ΔBT = difference in the bedtime between children and parents.

Bedtime was significantly later for parents in the separate bedroom group compared with the two subgroups of the bedroom sharing group (both p <* *0.05). However, there was no other intergroup/subgroup difference in sleep variables for children and parents. For participants in the bedroom sharing group, a shorter ΔBT was associated with a later bedtime and lower sleep efficiency of the children on weekdays (both p <* *0.05) and an earlier bedtime, longer sleep period, lower ESS score, higher sleep satisfaction and a paradoxically longer sleep latency for the parents (p <* *0.001, 0.01, 0.05, 0.05 and 0.01, respectively) (Table4).

**Table 4:** Influence of difference in bedtime between parents and children on sleep variables

Sleep variables	ΔBT in bedroom sharing group	
	Parents	Children
Weekday bedtime	0.882***	−0.383*
Weekday sleep end time	0.206	−0.166
Weekday sleep period	−0.493**	0.295
Sleep latency	−0.449**	0.099
Sleep efficiency	–	0.387*
Sleep problem	–	0.141
ESS score >10	0.321*	–
PSQI score >5	−0.033	–
Sleep satisfaction	−0.333*	–

ESS = Epworth Sleepiness Scale; PSQI = Pittsburgh Sleep Quality Index; ΔBT = difference in the bedtime between children and parents.

*p < 0.05, **p < 0.01, and ***p < 0.00, from Pearson's product–moment correlations or Spearman's rank order correlations.

## Discussion

In healthy Japanese 5-year-old children, the sleep end time of parents appeared to play a key role in regulating children's sleep patterns. For our current cohort, where bedroom sharing was common, co-sleeping was not associated with adverse sleep patterns of children or parents. However, children and parents going to sleep at a similar time were associated with unfavourable sleep patterns in both children and their parents. Further investigations are required to delineate specific characteristics of bedroom sharing and co-sleeping associated with unfavourable sleep patterns of children and parents.

### Sleep patterns of the children and parents

We observed that the sleep end time of the parents was linearly associated with the children's sleep variable scores. In our study population, the vast majority of the primary parents were mothers, where interactions of sleep patterns between the children and their parents might be relatively more prominent, because mothers' sleep habits are reported to have a stronger influence than fathers' 16. It is unclear whether children's chronotype mimics that of their parents in later developmental stages, including unfavourable trends such as eveningness. A study of early-teenage school children in Taipei showed that sleep patterns were not correlated between the children and their parents. However, several social factors in Taipei (e.g. schools generally open earlier than parents' work places, and children often have breakfast and walk to school on their own) should be considered 4. Future longitudinal studies should address developmental changes in the dependence of children's sleep behaviours on their parents, taking into account different cultural backgrounds.

### Influence of parental sleep disorders on their children's sleep patterns

In our current study, the high PSQI scores of parents did not affect sleep behaviour of the 5-year-old children. Furthermore, parents with a high ESS score appeared to have a paradoxically positive influence on their children's sleep patterns, such as the early bedtime and high sleep efficiency, a finding inconsistent with previous studies suggesting negative effects of parental stress on their children's sleep 1. In addition, a lower ESS score was associated with a shorter ΔBT (see the next paragraph for a detailed discussion). It is possible to explain that the parents with high ESS scores may be sleeping solitarily or may fall asleep much later than their children, potentially improving the sleep quality of the children. Further studies are required to confirm these findings.

### Difference in bedtimes between children and parents: influence on sleep patterns

There are marked cross-cultural variations in sleeping arrangements. A large-scale cross-sectional study reported that in Western countries, the most common behaviour for infants occurring at bedtime is falling asleep independently in their own bed (57%), whereas in Asian countries, this rate is substantially lower (4%) 2. Researchers have investigated the influence of co-sleeping on sleep quality and emotional development of children. When a mother and an infant co-sleep, levels of partner-influenced arousal can be significantly higher, resulting in reduced sleep quality 17. In addition, co-sleeping children may have difficulty getting back to sleep on their own 5,8. A study of school-aged children found an association between co-sleeping and tiredness 18. However, there can be emotional security benefits from increased sensory contact and proximity during the night between children and parents, such as reduction in night fears in children and fulfilment of the protective instinct in parents 19,20. Moreover, co-sleeping might contribute to children having significantly higher self-esteem, experiencing less guilt and anxiety, and exhibiting a feeling of satisfaction with life 21,22.

Our current results suggest that, for parents who share a bedroom with their children, falling asleep at a similar time as their children negatively affects their and their children's sleep patterns, which may account for a considerable portion of adverse events attributed to bedroom sharing and co-sleeping. Circumstances in which parents try to get their children to go to sleep and subsequently fall asleep themselves shortly thereafter may be associated with poor sleep quality in the parents and subsequently in their children. Interestingly, in this study, parents who fell asleep at a similar time as their children appeared to be unaware of their and their children's sleep problems, as shown in their high sleep satisfaction and low ESS scores and unelevated rates in their children's sleep problems. It is possible that such parents might secure enough sleep periods to compensate for the poor sleep quality. Interventions to better manage the bedtime of children and parents in co-sleeping families might improve sleep patterns of both children and their parents.

## Limitations

We were able to recruit only a limited number of participants for our current study, resulting in a lack of sufficient statistical power in identifying detailed interactions in sleep patterns between the children and their parents. Actigraphy was available only for the children. Future studies need to address simultaneous, objective evaluation of sleep variables in both children and their parents to facilitate direct comparisons between the two. In the vast majority of the participating families, children and parents shared a bedroom, and our findings might not be applicable to families in Western countries. Further research is required to investigate the influence of specific sleep arrangements associated with bedroom sharing and co-sleeping on the sleep patterns of children and their parents.

## Conclusions

Parents' sleep behaviour influenced the sleep schedule and sleep quality of the children. Parents' falling asleep at a similar time to their children appeared to be a risk factor for lowered sleep quality in both the children and their parents. To promote optimal sleep habits in young children, understanding parents' sleep patterns along with those of the children would be important. Studies that account for multiple factors, including detailed sleep arrangements and cultural backgrounds, may help improve the sleep quality of individuals who share a bedroom and/or co-sleep with children or parents.
